# Noninvasive Diagnosis of Splenosis Using Tc‐99m Heat‐Denatured Red Blood Cell Scintigraphy

**DOI:** 10.1002/ajh.70501

**Published:** 2026-09-09

**Authors:** Natacha Dewarrat, Katharina Auf der Springe, Francesco Grandoni, Rami Hajri, Mathilde Gavillet

**Affiliations:** ^1^ Service and Central Laboratory of Hematology, Department of Oncology and Department of Laboratories and Pathology Lausanne University Hospital (CHUV) and University of Lausanne (UNIL) Lausanne Switzerland; ^2^ Nuclear Medicine and Molecular Imaging Department Lausanne University Hospital Lausanne Switzerland; ^3^ Department of Diagnostic and Interventional Radiology Lausanne University Hospital and University of Lausanne Lausanne Switzerland

**Keywords:** hereditary spherocytosis, splenectomy, splenosis

A 23‐year‐old patient was referred to our out‐patient clinic for the investigation of abdominal masses discovered during a gynecological examination.

The patient's medical history was notable for hereditary spherocytosis treated by splenectomy 5 years earlier, resulting in complete resolution of anemia and, later, loss to hematological follow‐up. The blood count was consistent with the expected post‐splenectomy profile: hemoglobin 142 g/L (117–157 g/L) with reticulocytosis (122.8 × 10^9^/L; 20–120 × 10^9^/L), thrombocytosis 444 × 10^9^/L (150–350 × 10^9^/L). Haptoglobin was undetectable (< 0.1 g/L; 0.3–2 g/L) and the other hemolysis parameters (LDH, total bilirubin) were within the normal range. The blood smear examination demonstrated spherocytes, Howell‐Jolly bodies, polychromasia, and occasional erythroblasts. Neither peripheral blood smear nor flow cytometry provided evidence of circulating lymphoproliferative disease. A pelvic MRI showed well‐defined and contrast‐enhancing masses without signs of tissue invasion (Figure 1A,B,E), the largest measuring 44 mm. Following additional contrast‐enhanced CT evaluation (Figure 1D), the differential diagnosis included splenosis and lymphoma, and a biopsy was recommended.

**FIGURE 1:**
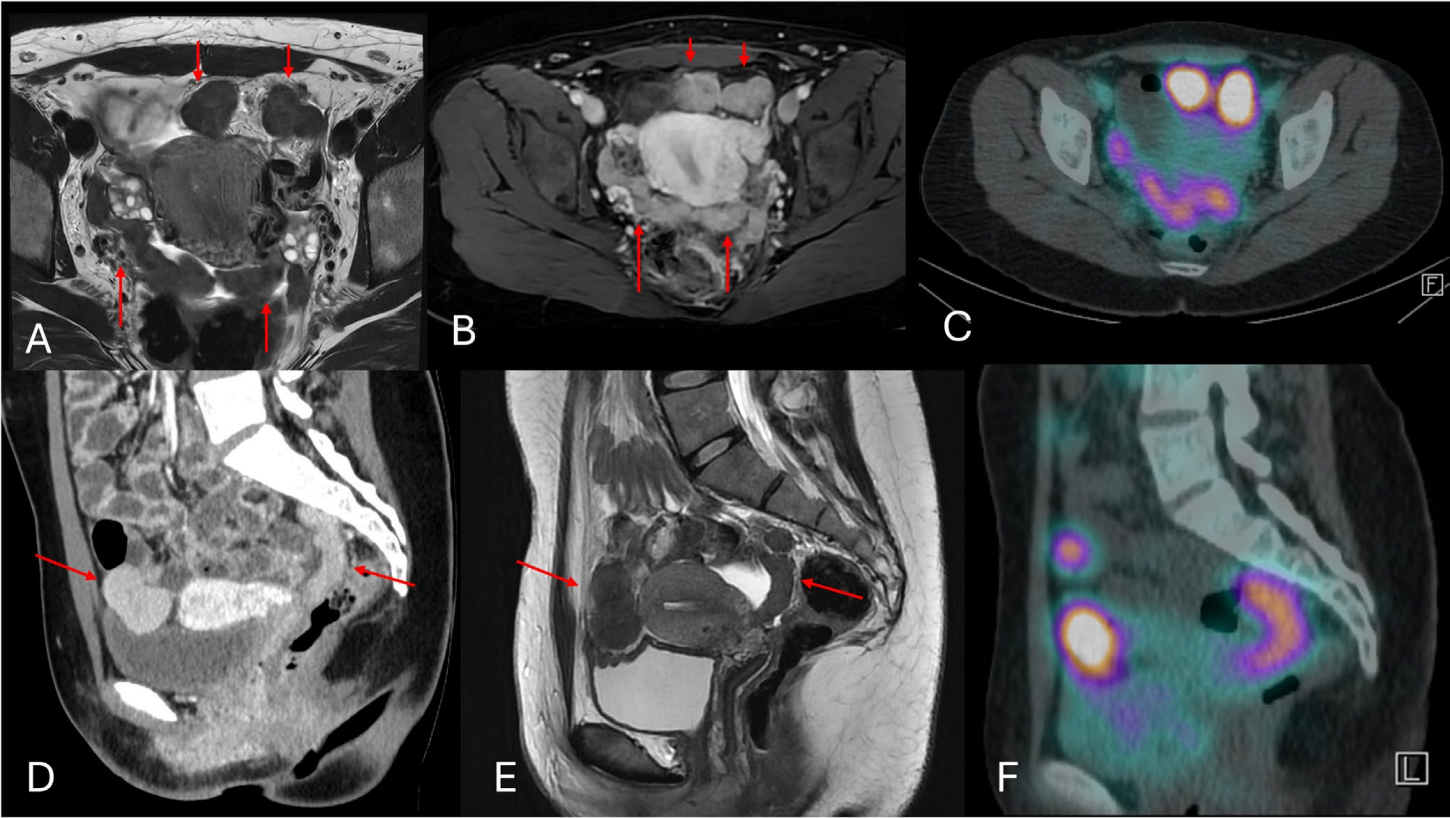
Representative images of the same nodules using different imaging techniques. (A) Axial T2‐weighted MRI demonstrating well‐defined peritoneal nodules (arrows) located in the prevesical space (superior arrows) and the pouch of Douglas (inferior arrows), and (B) contrast‐enhanced fat‐suppressed T1‐weighted MRI showing enhancement. (C) Axial fused 99mTc pertechnetate SPECT/CT demonstrating tracer‐avid peritoneal nodules (arrows) in the prevesical space and the pouch of Douglas. (D–F) Corresponding sagittal contrast‐enhanced CT (D), T2‐weighted MRI (E), and fused 99mTc pertechnetate SPECT/CT (F) demonstrate the same well‐defined peritoneal nodules (arrows) in the prevesical space and the pouch of Douglas.

At this stage of the investigations, ectopic splenic tissue was deemed unlikely given to the presence of numerous Howell body bodies as well as persistent thrombocytosis. Conversely, lymphoma could neither be confirmed nor excluded. Although tissue biopsy represents the diagnostic gold standard to elucidate the nature of the masses, a percutaneous transabdominal approach carried a substantial risk of bleeding due to their anatomical location.

The patient subsequently underwent selective spleen scintigraphy using technetium‐99 m‐labeled, heat‐damaged red blood cells with Single‐Photon Emission Computed Tomography/Computed Tomography (SPECT/CT). This well‐established technique is available in specialized nuclear medicine centers worldwide equipped with radio‐pharmaceutical facilities capable of processing blood samples. Following intravenous administration of stannous pyrophosphate to facilitate intracellular trapping of technetium within red blood cells, a small peripheral blood sample (typically around 5 mL) is collected. The red blood cells are then isolated by centrifugation, heat damaged (e.g., heated for 20 min in a water bath at 49.5°C/121°F, stirring every 5 min) and incubated with technetium‐99 m pertechnetate. Subsequently, the resulting radiolabeled red blood cells are reinjected intravenously [1]. Owing to the spleen's physiological filtering function, the damaged labeled erythrocytes selectively accumulate within splenic tissue. In our patient, SPECT/CT demonstrated intense tracer uptake in multiple peritoneal nodules, confirming the diagnosis of splenosis (Figure 1C,F).

Notably, ectopic splenic tissue, due to its limited mass, disorganized architecture, and variable vascularization, is generally less effective than a normal spleen at filtering erythrocytes containing Howell–Jolly bodies and at sequestering platelets. Consequently, the persistence of Howell–Jolly bodies and thrombocytosis does not exclude the presence of splenosis.

This case highlights the value of 99mTc‐labeled heat‐damaged red blood cell SPECT/CT in establishing a confident, noninvasive diagnosis of splenosis, thereby avoiding unnecessary biopsy and its associated risk of bleeding.

## Funding

The authors have nothing to report.

## Ethics Statement

The authors have nothing to report.

## Consent

Written informed consent was obtained from the patient for publication of the clinical information and images included in this article.

## Conflicts of Interest

The authors declare no conflicts of interest.

## Data Availability

Data sharing not applicable to this article as no datasets were generated or analyzed during the current study.
